# Synthesis and characterization of thiolated hexanoyl glycol chitosan as a mucoadhesive thermogelling polymer

**DOI:** 10.1186/s40824-018-0137-7

**Published:** 2018-09-26

**Authors:** Ik Sung Cho, Hye Min Oh, Myeong Ok Cho, Bo Seul Jang, Jung-Kyo Cho, Kyoung Hwan Park, Sun-Woong Kang, Kang Moo Huh

**Affiliations:** 1 Department of Organic Materials Engineering, Chungnam National University, 99 Daehak-ro, Yuseong-gu, Daejeon, 34134 Republic of Korea; 2 Predictive Model Research Center, Korea Institute of Toxicology, 141, Gajeong-ro, Yuseong-gu, Daejeon, 34114 Republic of Korea; 3 ezlab, 120, Heungdeokjungang-ro, Giheung-gu, Yongin-si, Gyeonggi-do 16950 Republic of Korea; 4 Human and Environmental Toxicology Program, University of Science and Technology, 217, Gajeong-ro, Yuseong-gu, Daejeon, 34113 Republic of Korea

**Keywords:** Hexanoyl glycol chitosan, Chemical modification, Mucoadhesive polymer, Rheological measurements, Thermogelling property, Thiolation

## Abstract

**Background:**

Mucoadhesive polymers, which may increase the contact time between the polymer and the tissue, have been widely investigated for pharmaceutical formulations. In this study, we developed a new polysaccharide-based mucoadhesive polymer with thermogelling properties.

**Methods:**

Hexanoyl glycol chitosan (HGC), a new thermogelling polymer, was synthesized by the chemical modification of glycol chitosan using hexanoic anhydride. The HGC was further modified to include thiol groups to improve the mucoadhesive property of thermogelling HGC. The degree of thiolation of the thiolated HGCs (SH-HGCs) was controlled in the range of 5–10% by adjusting the feed molar ratio. The structure of the chemically modified polymers was characterized by ^1^H NMR and ATR-FTIR. The sol-gel transition, mucoadhesiveness, and biocompatibility of the polymers were determined by a tube inverting method, rheological measurements, and in vitro cytotoxicity tests, respectively.

**Results:**

The aqueous solution (4 wt%) of HGC with approximately 33% substitution showed a sol-gel transition temperature of approximately 41 °C. SH-HGCs demonstrated lower sol-gel transition temperatures (34 ± 1 and 31 ± 1 °С) compared to that of HGC due to the introduction of thiol groups. Rheological studies of aqueous mixture solutions of SH-HGCs and mucin showed that SH-HGCs had stronger mucoadhesiveness than HGC due to the interaction between the thiol groups of SH-HGCs and mucin. Additionally, we confirmed that the thermogelling properties might improve the mucoadhesive force of polymers. Several in vitro cytotoxicity tests showed that SH-HGCs showed little toxicity at concentrations of 0.1–1.0 wt%, indicating good biocompatibility of the polymers.

**Conclusions:**

The resultant thiolated hexanoyl glycol chitosans may play a crucial role in mucoadhesive applications in biomedical areas.

## Background

Mucoadhesive polymers have been extensively investigated as pharmaceutical formulations for drug delivery systems due to their many potential advantages, such as prolonged residence time, improved drug bioavailability, and reduced administration frequency [1]. Therefore, many researchers have developed mucoadhesive polymers as drug delivery carriers via various administration routes, including ocular, nasal, gastrointestinal and vaginal routes [2–5].

Recently, thermogelling polymers that show a thermosensitive sol-gel transition in aqueous media have received much attention for mucoadhesive drug delivery due to their potential for easy administration and prolonged active residence time on the mucosal surface [6]. A sol-gel transition property may allow administration in a solution formulation (by spraying, dropping, injecting, etc.) below the transition temperature, ensuring complete spreading on the mucous layer. After application, the body temperature causes the solution to undergo quick gelation, which can subsequently stabilize the formulation and overcome the early removal mechanism of the formulation from the mucosa, prolonging the residence time of the loaded drug at the administration site [7].

Most typical thermogelling polymers, such as PEG/PPG and PEG/PLA block copolymers, demonstrate poor bioadhesion and low physical stability although they require a high concentration of polymer for thermogelation, limiting their practical application as mucoadhesive formulations. To overcome their limitations, Yuan et al. developed a rectal gel formulation based on a mixture of poloxamer 407 and sodium alginate/hydroxypropyl methylcellulose (HPMC) as a thermogelling component and a mucoadhesive component, respectively [8]. Wu et al. prepared a thermosensitive hydrogel as a nasal drug delivery system using a mucoadhesive polysaccharide such as chitosan [9]. However, since the polymer alone cannot demonstrate any thermosensitivity, the chemical modification and the additional use of salts or additives such as α,β-glycerophosphate (α,β-GP) were needed for thermogelation.

A new generation of mucoadhesive polymers, designated thiolated polymers, that contain thiol functional moieties has been developed. Due to the presence of thiol groups on the polymer backbone, thiolated polymers have the ability to form a covalent disulfide bond with the mucous layer, leading to enhanced mucoadhesive properties [10, 11]. The mechanism is based on thiol/disulfide exchange reactions and an oxidation process between the reactive thiol groups of the mucoadhesive polymer and the cysteine-rich subdomains of the mucin glycoproteins [12]. Therefore, various thiomers as mucoadhesive polymers have been developed, such as thiolated xyloglucan [13], alginate-cysteine conjugate [14], thiolated chitosan [15], thiolated gelatin [16], thiolated poly(aspartic acid) [17], and thiolated silicone oil [18]. However, these polymers do not have thermogelling properties, and they require a long time or the addition of chemicals for stable hydrogel formation [17, 19].

Chitosan, one of the polysaccharides, is known to be biocompatible, biodegradable, and mucoadhesive [20–22]. Because of its many advantages, chitosan has been extensively investigated for pharmaceutical, cosmetics, biomedical, and biotechnological applications [23]. However, one of the major disadvantages for its use as a biomaterial is the poor water solubility in physiological conditions [24]. Glycol chitosan, a water-soluble chitosan derivative, is easily soluble in aqueous media regardless of the pH and has free amine groups available for further chemical modifications. Additionally, its low toxicity and good biocompatibility makes it more suitable for biomedical applications [25].

Recently, new classes of polysaccharide-based thermogelling polymers, acyl glycol chitosans that were prepared by the *N*-acylation of glycol chitosan, have been reported by our group, and their basic properties have been studied for various biomedical applications. Their promising properties, such as biocompatibility, biodegradability, and thermoreversible sol-gel transition behavior (even at the low range of concentrations from 3 to 7 wt%), could make the acyl glycol chitosans useful as new potential biomaterials for various biomedical applications, including injectable drug delivery systems and cell/tissue engineering. One of the acyl glycol chitosans, acetylated glycol chitosan, was evaluated as a mucoadhesive thermogelling polymer to develop a vaginal delivery hydrogel formulation of progesterone [26]. The results showed that the hydrogel formulation retained many characteristics useful for an effective vaginal delivery system and could be a promising alternative to current mucoadhesive formulations. However, these acyl glycol chitosans have mucoadhesive properties based on only the noncovalent bond formation such as hydrogen bonds, ionic interaction, and polymer chain entanglement between the polymer and the mucous layer, and thus may provide a limited range of mucoadhesiveness [27].

The objective of the present study is to develop a glycol chitosan-based thermogelling polymer with enhanced mucoadhesive properties via thiolation. Here, new mucoadhesive thermogelling polymers, thiolated hexanoyl glycol chitosans (SH-HGCs), were synthesized by a series of *N*-hexanoylation and *N*-thiolation reactions of glycol chitosans. SH-HGCs with different degrees of thiolation were synthesized and characterized by ^1^H NMR and ATR-FTIR measurements. Their thermogelling and mucoadhesive properties were evaluated and compared with HGC by rheological measurements. Three kinds of in vitro cytotoxicity tests were performed to investigate the potential of SH-HGCs for biomaterials application by an MTT assay using HeLa cells and human fibroblasts, a direct contact method using epithelial cells, and a live and dead assay using epithelial cell aggregates.

## Methods

### Materials

Glycol chitosan (GC, DP ≥ 200) and hexanoic anhydride (97%) were purchased from WAKO (Japan) and Sigma-Aldrich (India), respectively. 3-Mercaptopropionic acid, 1-ethyl-3-(3-dimethylaminopropyl)carbodiimide hydrochloride (EDC), *N*-hydroxysuccinimide (NHS), and mucin from porcine were purchased from Sigma-Aldrich (St. Louis, MO, USA). Acetone, methanol and ethanol were supplied from Samchun Chemical (Korea).

### Synthesis of hexanoyl glycol chitosan

Hexanoyl glycol chitosan (HGC) was synthesized by the *N*-acylation of GC under mild conditions. Briefly, 3 g of GC was dissolved in 375 mL of distilled water and diluted with 375 mL of methanol. A predetermined amount (1.029 mL) of hexanoic anhydride was added to a GC solution under magnetic stirring. After continuous stirring at room temperature for 24 h, the polymer was collected by precipitation in acetone. The polymer was then dialyzed against distilled water for 2 days using a dialysis membrane (molecular weight cut-off, 12–14 KDa), followed by lyophilization. Lyophilization procedure is as follows: the obtained aqueous polymer solution was frozen in liquid nitrogen for 15 min. Subsequently, a flask containing the solution was submitted to lyophilizer (ISFD-8512, iSBio, South Korea) for 3 days. The temperature of the condenser was − 85 °C and the pressure was 5 mTorr. The lyophilized polymers were finally collected and kept in the refrigerator (below 4 °C) until use.

### Synthesis of thiolated hexanoyl glycol chitosans (SH-HGCs)

Thiolated hexanoyl glycol chitosans (SH-HGCs) were synthesized by thiolation of HGC. Briefly, 0.5 g of HGC was dissolved in 100 mL of distilled water. To activate the carboxylic acid of 3-mercaptopropionic acid, predetermined amounts of 3-mercaptopropionic acid (0.009–0.019 mL), EDC (60 mg), and NHS (60 mg) were added to 10 mL MES buffer, and the pH was adjusted to 5.2 by adding 0.1 M HCl. After 2 h, the activated 3-mercaptopropionic acid solution was added to an HGC solution. The reaction was then carried out at room temperature under stirring for 24 h. The resultant polymers were then dialyzed against distilled water for 2 days using a dialysis membrane (molecular weight cut-off, 12–14 KDa) and lyophilized with the same conditions as previously mentioned.

### Characterization of SH-HGCs

SH-HGCs were characterized by ^1^H NMR spectroscopy using an AVANCE III 600 spectrometer (BRUCKER, Germany) operating at 600 MHz. The polymer samples were dissolved in D_2_O at 1.0 wt%. The D_2_O peak at δ 4.65 was used as a reference peak. To confirm the polymer composition, ATR-FTIR spectra of GC and HGCs were recorded using a Nicolet iS 5 (Thermo Scientific, USA). The ATR-FTIR analysis was performed with 16 scans at a resolution of 4 cm^− 1^ over a frequency range of 4000–750 cm^− 1^.

### Thermogelling properties

The sol–gel transition temperatures of HGC and SH-HGCs were determined using a tube inverting method with a temperature increase of 0.5 °С/min. The polymer solutions (4 wt%, 1 mL) were prepared by dissolving HGC in phosphate-buffered saline (PBS, pH 7.4) at 4 °С in a 5-mL vial. The sol–gel transition temperature was determined over 1 min in the inverted tube [28]. Each data point is an average of three measurements with the standard deviation (mean ± SD). The sol–gel transition phase diagram obtained from this method is known to have a precision of ±1 °С [29]. Sol–gel transition temperatures of the aqueous HGC and SH-HGC solutions were also investigated by a rotating rheometer (TA Instruments, AR 1500ex). The aqueous solutions of GC, HGC and SH-HGCs were placed between parallel plates with a 20 mm diameter and a gap of 1 mm. The frequency was optimized to 1 Hz, as determined using a frequency sweep experiment. A constant stress of 25 Pa was used for the measurement.

### Mucoadhesion analysis

Rheological measurement is one of the most common methods to evaluate the mucoadhesion of polymers [30]. To determine the mucoadhesive properties of HGC and SH-HGCs, an HGC-mucin mixture and an SH-HGC-mucin mixture were prepared in PBS (pH 7.4). The final concentration of mucin, HGC, or SH-HGCs in the PBS solutions was 4 wt%. Rheological evaluations were carried out at 25 °С and 37 °С using a MARS-40 (Thermo Scientific, USA) with a parallel plate (20 mm) and a gap of 1 mm. Polymer samples were loaded on the rheometer platform and equilibrated at 25 °С or 37 °С for 1 min before rheological measurement. A frequency sweep analysis was performed from 0.1 to 10 Hz to determine the storage modulus (*G*`). All samples were prepared in triplicate (*n* = 3). The mean values of the storage modulus for each sample were taken from the frequency sweep spectra. The absolute synergism parameter (△*G*`) is a storage modulus component showing the interactions among polymers and mucins [31]. The following equation was used to calculate △*G*` [32]:

$$ \Delta {G}^{`}={G}_{\mathrm{m}\mathrm{ix}}^{`}-{G}_{\mathrm{p}}^{`}-{G}_{\mathrm{m}}^{`}, $$

where *G*’_mix_, *G*’_p_, and *G*’_m_ are the storage moduli of the mixture, polymers, and mucin, respectively. The elastic modulus of the mucin solution can be removed from the equation since the elastic modulus of the mucin dispersion was negligible [30, 31]. Therefore, △*G*` was determined from the following equation:

$$ {\varDelta G}^{`}={G}_{\mathrm{mix}}^{`}-{G}_{\mathrm{p}}^{`}. $$

### Cytotoxicity tests by MTT and direct contact method

The in vitro cytotoxicity of SH-HGCs was evaluated using a 3-(4,5-dimethylthiazol-2-yl)-2,5-diphenyl tetrazolium bromide (MTT, sigma) assay. HeLa cells and human lung fibroblasts were seeded on 96-well tissue culture plates (Corning, Saint Louis, MO, USA) at a density of 5 × 10^3^ cells/well and incubated for 24 h at 37 °С in the presence of 5% CO_2_. The culture medium was then replaced by various concentrations of SH-HGCs (0, 0.1, 0.5, and 1.0 wt% in culture medium). After incubation for 24 h, the MTT solution (20 μl, 5 mg/mL in PBS) was added to each well, and the cells were incubated for 2 h at 37 °С. After removing the MTT solution, 150 μL of dimethyl sulfoxide (DMSO, Sigma) was added to dissolve the formazan crystals. The absorbance of each well was measured by a microplate reader (SpectraMax M3, Molecular devices, Sunnyvale, CA, USA) at 540 nm.

For the direct contact test, the cultured cells (human conjunctiva epithelial cells; Korean Cell Line Bank, Seoul, Korea) were plated at 2 × 10^5^ cells/well and preincubated for 24 h. After 24 h, specimen films (1 × 1 cm^2^) were placed on the centers of the wells where a confluent monolayer of the cells had formed, and the cells were cultured for 24 h. After removing the specimens from each well, the wells were washed with PBS and stained with 0.2% crystal violet solution. The percentage of the detached area affected by the cytotoxic specimen was measured using an image analysis system (ImageJ, a public domain, Java-based, image-processing software program developed by the National Institutes of Health). The results were interpreted by the grade of reactivity zone (Table 1). The PU-ZDEC film (Hatano Research Institute, Kanagawa, Japan) served as the positive control.

**Table 1:** Reactivity grades for direct contact cytotoxicity [39]

Grade	Reactivity	Description of reactivity zone
0	None	No detectable zone around or under specimen
1	Slight	Zone limited to area under specimen
2	Mild	Zone extends less than 0.5 cm beyond specimen
3	Moderate	Zone extends 0.5–1.0 cm beyond specimen
4	Severe	Zone extends greater than 1.0 cm beyond specimen but does not involve entire dish

### Viability assay of multicellular epithelial cells on SH-HGCs

For the formation of multicellular aggregates of epithelial cells (human conjunctiva epithelial cells), the cells were plated at densities of 5 × 10^4^ cells/well (12-well dish; ULA plate, Corning, Saint Louis, MO, USA) and incubated for 1 day. Next, epithelial cell aggregates were transferred to SH-HGC coated plates and cultured for 1 day. The viability of multicellular aggregates was determined with a live/dead assay kit (Abcam, Cambridge, United Kingdom). The culture medium was exchanged with a staining solution, and the samples were incubated for 15 min at 37 °C and examined via fluorescence microscopy (DMi8; Leica, Heerbrugg, Germany).

### Statistical analysis

Statistical analysis was performed using an Origin pro version 8 software package (OriginLab Corp., MA, USA) to determine the significant difference. The experimental data are presented as the mean ± standard deviation and were analyzed with one-way analysis of variance (one-way ANOVA). A value of *p < 0.05 was considered statistically significant.

## Results

### Synthesis and characterization of SH-HGCs

**Fig. 1:**
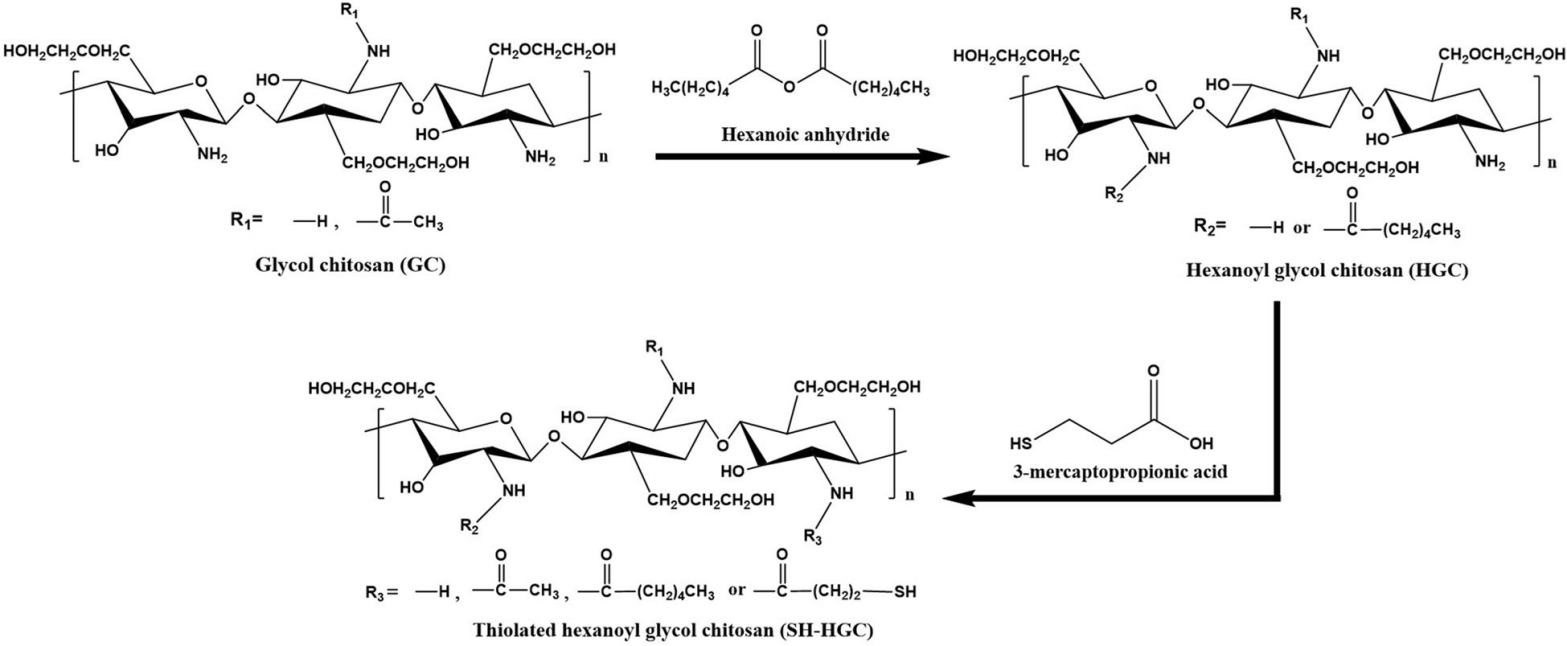
Synthetic scheme of thiolated hexanoyl glycol chitosan

**Fig. 2:**
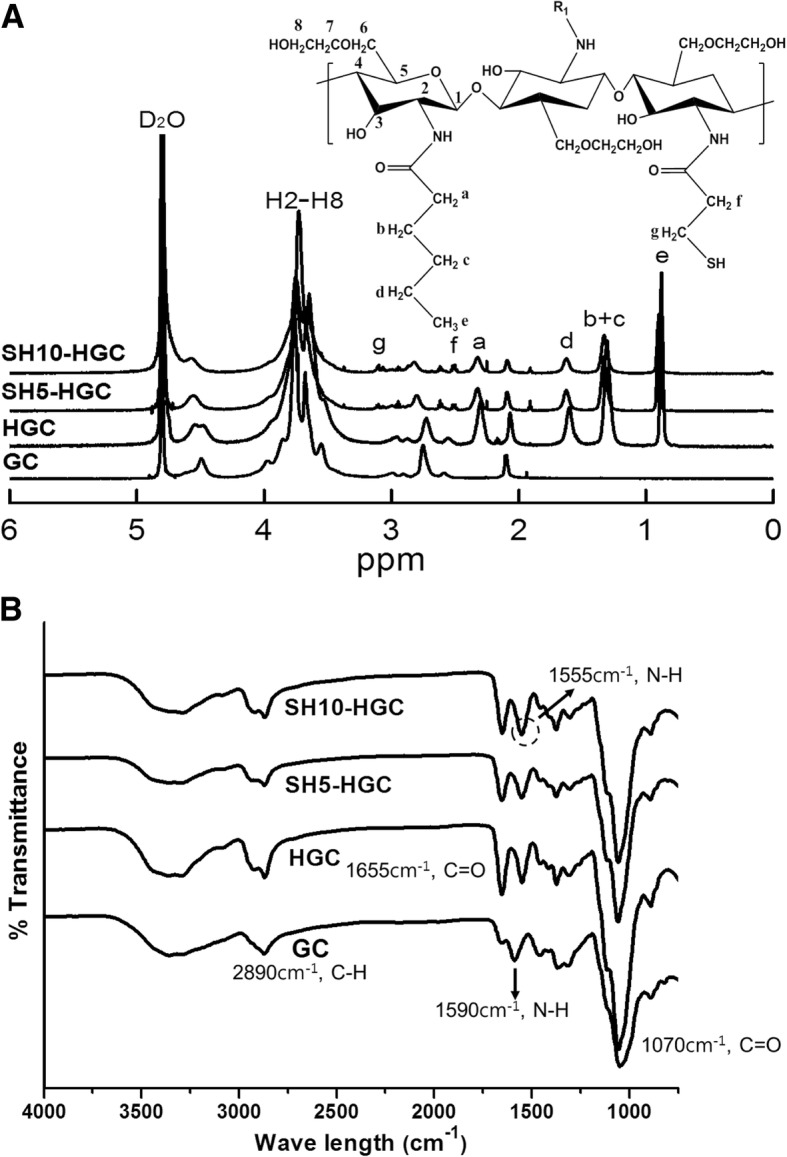
(**a**) ^1^H NMR spectroscopy and (**b**) ATR-FTIR spectra of GC, HGC, and SH-HGC

Various SH-HGCs with different degrees of thiolation were synthesized from glycol chitosan using a two-step reaction procedure under mild conditions (Fig. 1). In the first step of the reaction, the amino groups of glycol chitosan were reacted with hexanoic anhydride to form HGC. In the second step, the obtained HGC was further modified by reacting the residual amine groups with the carboxylic acids of 3-mercaptopropionic acid to form SH-HGCs. The chemical compositions of the HGC and SH-HGCs were confirmed by ^1^H NMR measurements. The ^1^H NMR spectra of GC, HGC, and SH-HGCs are shown in Fig. 2a. The D_2_O peak was used as a reference peak at 4.65 ppm. The overlapped peaks at 3.2–4.0 ppm contributed to the protons of the glucopyranosyl rings at positions 2–8 (H-2 through H-8). The peak at 2.65 ppm arose from the protons of the primary amine residues. The peak at 2.0 ppm was assigned to the methyl protons of the acetyl group in GC. The new proton peaks at 0.8, 1.2, 1.5, and 2.2 ppm were assigned to -C*H*_3_, -C*H*_2_–C*H*_2_–CH_3_, -CO-CH_2_-C*H*_2_-, and -CO-C*H*_2_- of the hexanoyl groups, respectively. Based on these assignments, the degree of hexanoylation (DH) of HGC was calculated as approximately 33% by comparing the integrated signal area of the protons of the glucopyranosyl ring with that of the hexanoyl groups. As shown in Fig. 2a, thiolation was successfully achieved, as evidenced by the peak arising at 3.1 and 2.5 ppm due to methylene protons of 3-mercaptopropionic acid residues in SH-HGCs. The degree of thiolation was calculated from the relative integration area of the methyl protons at 2.5 ppm compared with that of the glucopyranosyl ring protons at 3.2–4.0 ppm.

The chemical structures of GC, HGC, and SH-HGCs were also confirmed by ATR-FTIR (Fig. 2b). The broad band at 3400 cm cm^− 1^ was assigned to the stretching vibration of the hydroxyl groups, which overlapped with the N-H stretching vibrations in the same region. The absorption peak at 2900 cm^− 1^ was attributed to the –CH_2_- groups. The absorption peak at 1596 cm^− 1^ was ascribed to the amino bending vibration of GC. The presence of absorption bands at 1655 cm cm^− 1^ and 1555 cm cm^− 1^ corresponded to the carbonyl stretching and the amide II bending vibration of HGCs, respectively. The disappearance of the amino vibration band at 1596 cm cm^− 1^ and the appearance of the amide II band at 1555 cm cm^− 1^ after hexanoylation indicated that the HGCs were successfully synthesized [33]. Additionally, the increase in the peak intensity at 1555 cm^− 1^ relative to the peak at 1655 cm^− 1^ indirectly supports the thiolation of HGCs. The above ^1^H NMR and ATR-FTIR results confirmed that the chemical modifications, hexanoylation and thiolation, of GC were successfully accomplished. SH-HGCs with two different degrees of thiolation were synthesized by adjusting the feed molar ratio of 3-mercaptopropionic acid as shown in Table 2 (yield: 79–85%). The degree of thiolation (DT) of the SH-HGC could be tuned from 5.1 to 10.9% by controlling the feed molar ratio of the 3-mercaptopropionic acid (Table 2).

**Table 2:** Chemical data for the SH-HGC

Samples	Feed molar ratio^a)^	DT^b)^ (%)	Yield (%)
SH5-HGC	0.10	5.1 ± 0.5	85
SH10-HGC	0.20	10.9 ± 0.4	79

^a)^Feed molar ratio of 3-mercaptopropionic acid to glucosamine residue of GC

^b)^Degree of thiolation (DT) determined by the peak integration of ^1^H NMR

### Thermosensitive sol-gel transition

**Fig. 3:**
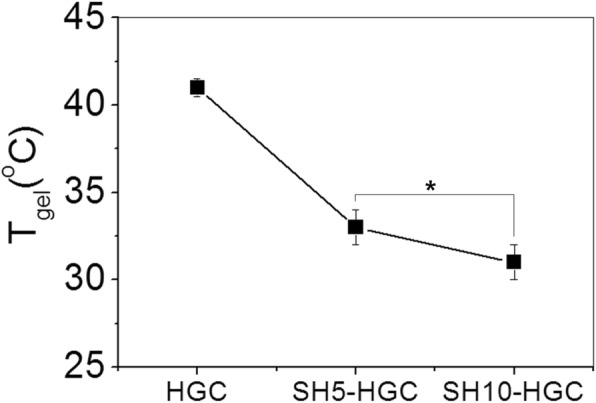
Sol-gel transition of HGC and SH-HGCs measured by the tube inverting method (*n* = 3, *p < 0.05)

The thermosensitive sol-gel transition properties of HGC and SH-HGCs were investigated by a tube inverting method. An aqueous solution of 4 wt% HGC underwent a phase transition from a flowing liquid (sol) to a non-flowing (gel) as the temperature increased. The SH-HGCs also showed a sol-gel phase transition, but their gelation temperatures (T_gel_) were observed at a lower temperature range relative to that of HGC. As shown in Fig. 3, the T_gel_ values of HGC, SH5-HGC, and SH10-HGC were observed at 41 ± 0.5, 34 ± 1, and 31 ± 1 °С, respectively.

**Fig. 4:**
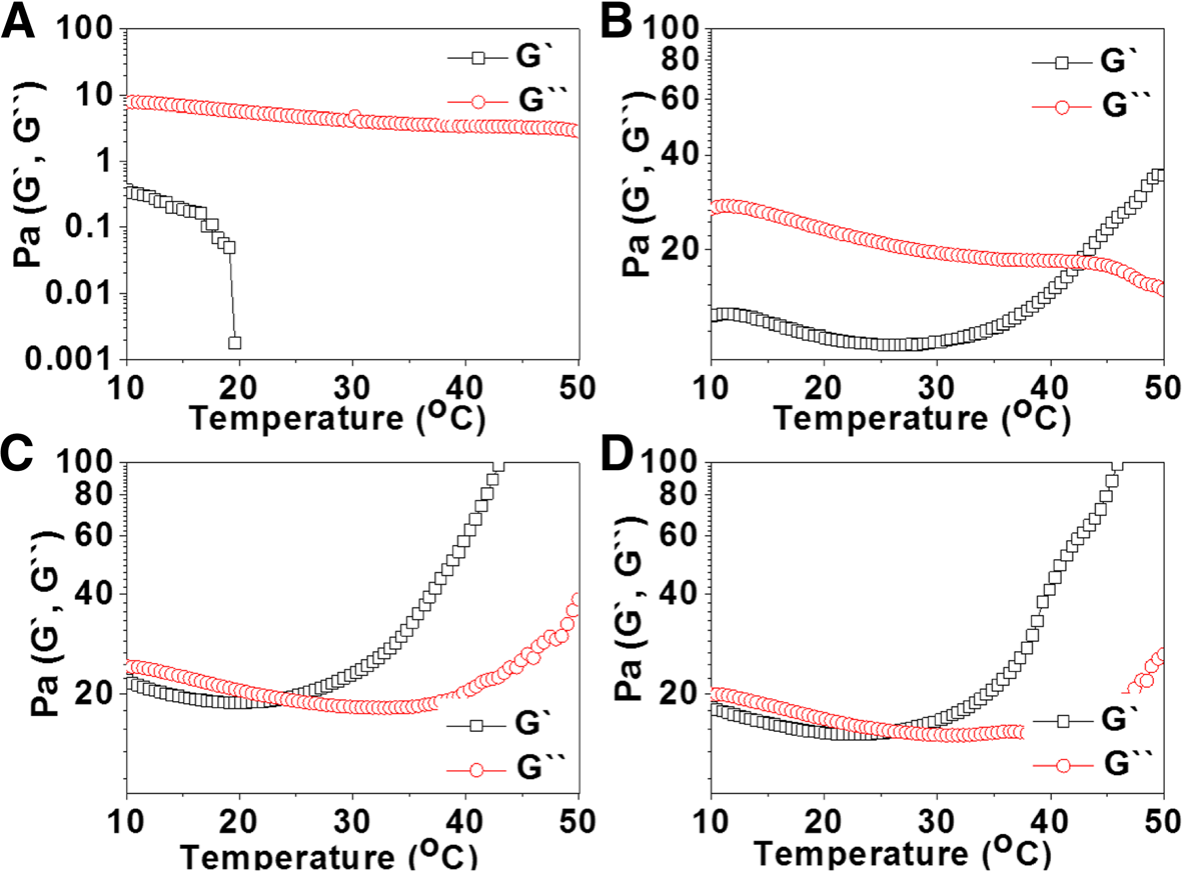
Temperature-dependent rheological behavior of the aqueous solutions (4 wt%) of (**a**) GC, (**b**) HGC, (**c**) SH5-HGC, and (**d**) SH10-HGC

Rheological studies were also carried out to determine the viscoelastic properties of HGC and SH-HGCs as a function of temperature. Fig. 4 shows the changes in the storage modulus (G`) and loss modulus (G``) of GC, HGC, SH5-HGC, and SH10-HGC (4 wt%, PBS) as a function of temperature. In case of GC, G`` was continuously higher than G` in the experimental temperature range from 10 to 50 °С. In case of HGC and SH-HGCs, at the initial temperature range (below T_gel_), G` was lower than G``; however, as the temperature increased above T_gel_, the G` of HGC and SH-HGCs increased rapidly, leading to a crossover with G`` at a certain temperature, T_gel_, indicating the sol-gel phase transition of the aqueous polymer solution. Among the GC derivatives, SH10-HGC showed the largest increase in the G` values.

### Mucoadhesion evaluation of thermogel

**Fig. 5:**
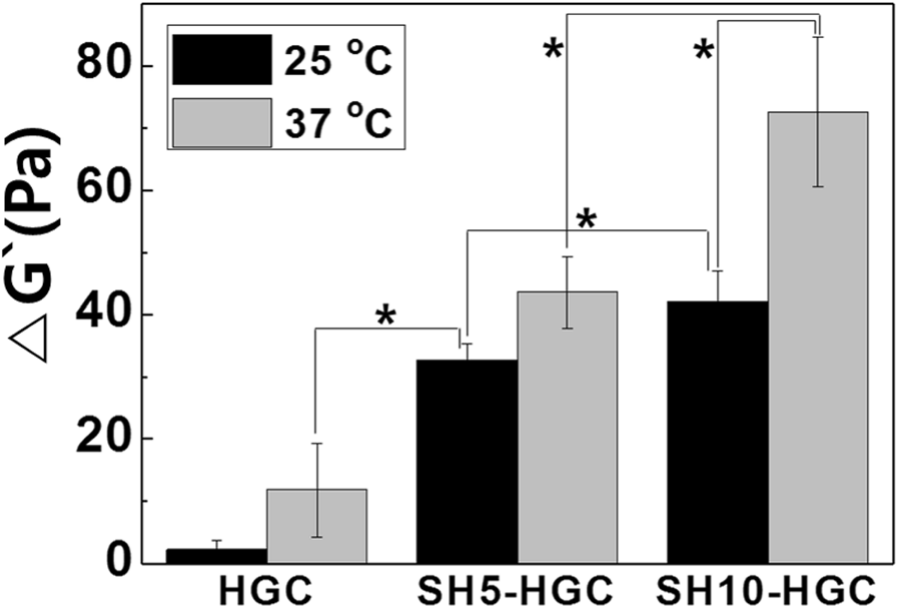
△G` of HCG and SH-HGC at 25 and 37 °C. The mucoadhesive properties of the gels are estimated by measuring △G` (*n* = 3, *p < 0.05)

By a rheological analysis, mucoadhesion of theromogels was evaluated. As shown in Fig. 5, the introduction of mucins affected the △G` values of the HGC, SH5-HGC, and SH10-HGC depending on temperature (25 °С, 37 °С). Rheological analysis of each polymer demonstrated a positive rheological synergism parameter (△G` > 0). The SH-HGCs showed a higher △G` relative to the HGC. The highest △G` was observed from SH10-HGC. We also observed temperature-dependent mucoadhesive properties of the hydrogels when comparing △G’s at 25 and 37 °С. As shown in Fig. 5, almost polymeric solutions showed higher △G’s at 37 °С than at 27 °С.

### In vitro biocompatibility

**Fig. 6:**
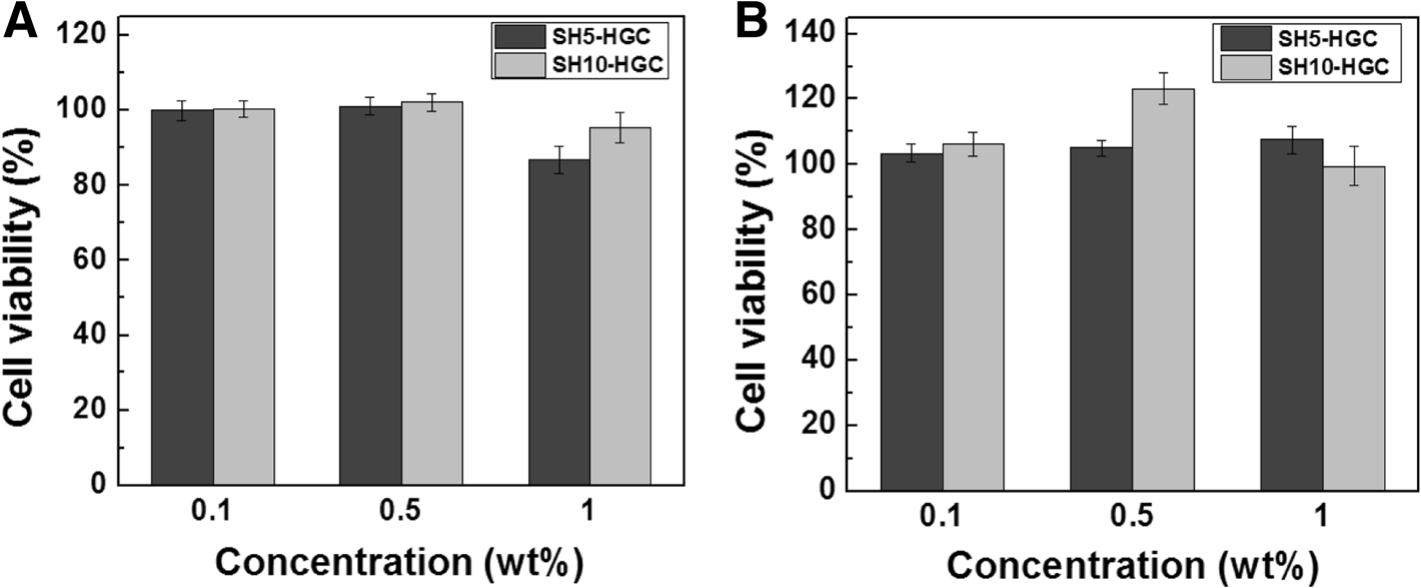
Viability of (**a**) HeLa cells and (**b**) human fibroblasts at various concentrations of SH5-HGC and SH10-HGC dilution medium

The cytotoxicity of the SH-HGCs was estimated by an MTT assay using HeLa cells and human fibroblasts as shown in Fig. 6. The cytotoxicity was determined with various concentrations of the polymers and degrees of thiolation in the SH-HGCs after 24 h of incubation. The SH5-HGC and SH10-HGC showed low cytotoxicity at various concentrations (0.1–1.0 mg/ml) for HeLa cells and human fibroblasts.

**Fig. 7:**
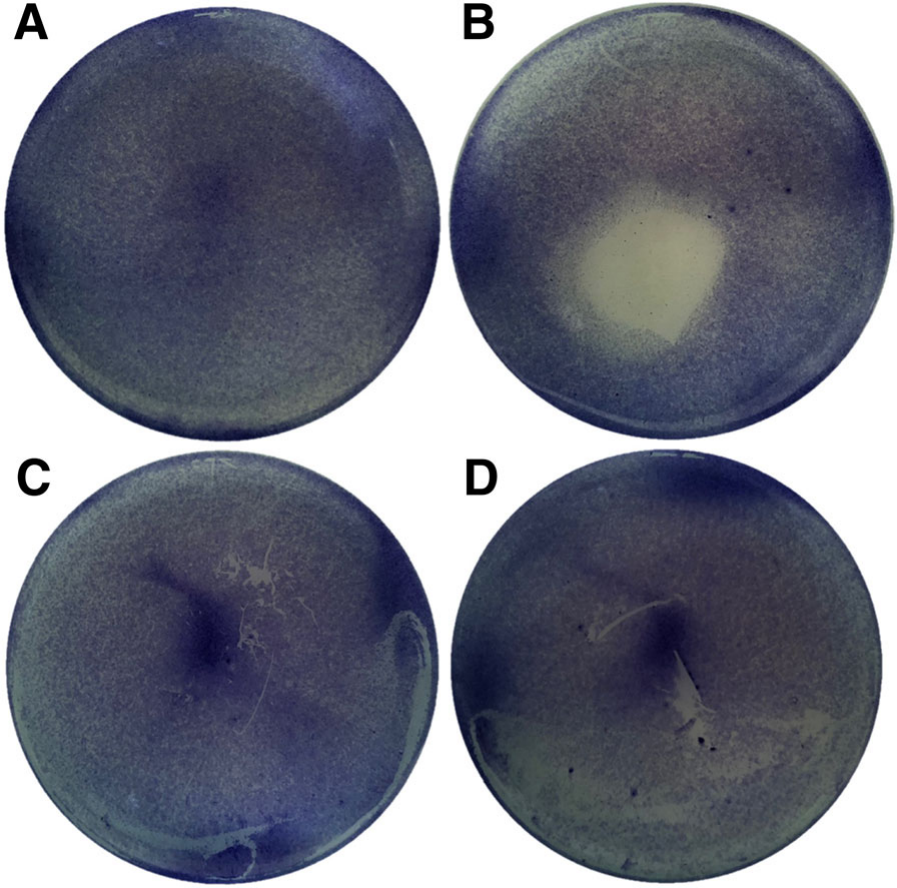
Direct contact cytotoxicity assay: (**a**) non-treatment, (**b**) PU-ZDEC, (**c**) SH5-HGC (5 wt%), (**d**) SH10-HGC (5 wt%)

Additionally, the cytotoxicity of SH-HGCs was evaluated by the direct contact method using epithelial cells. In the direct contact test, the size of the reactivity zone in the well was observed. The cells cultured under and around the PU-ZDEC were detached from the culture dish and produced a round-shaped cell-free zone on the plate by the PU-ZDEC film. In contrast, no cytotoxic regions were detected for the SH5-HGC and SH10-HGC hydrogels or the non-treatment group (Fig. 7).

**Fig. 8:**
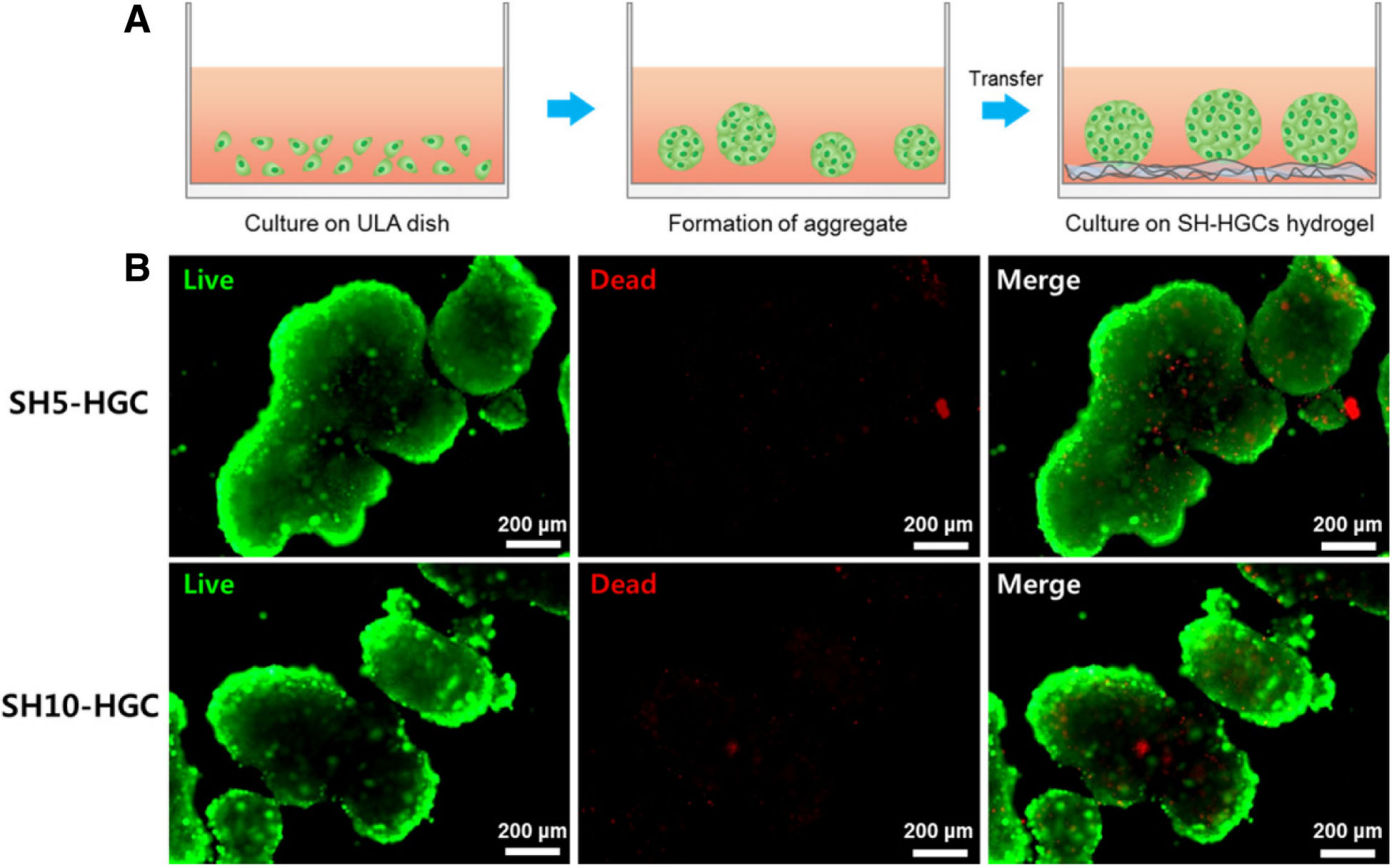
(**a**) Schematic illustration of aggregates forming process for live/dead assay and (**b**) live/dead assay of epithelial cell aggregates on HS5-HGC and HS10-HGC hydrogel at day 1

To determine whether multicellular aggregates adhering on SH-HGCs hydrogels were viable, we performed live and dead assays through staining with a live/dead assay kit on day 1. The epithelial cells were plated on a ULA culture dish. The multicellular aggregates were formed within 24 h on the ULA dish. When multicellular aggregates in the ULA dish for 1 day were transferred to the SH-HGC coated dish, the multicellular aggregates adhered on the surface of hydrogels. In addition, reasonable numbers of cells in aggregate were viable (Fig. 8). We could not find a difference between the cells on the SH5-HGC and SH10-HGC hydrogels. These results indicate that SH-HGCs may not affect the viability of the mucosa.

## Discussion

To develop a new mucoadhesive thermogelling polymer for potential biomedical applications, glycol chitosan was modified by a series of hexanoylation and thiolation reactions to give SH-HGCs. In our previous studies, the thermosensitive HGC was found to be useful for ocular delivery formulation by prolonging the retention time on the preocular surface and thus enhancing ocular bioavailablility [33]. Based on the promising physicochemical properties of HGC, we here synthesized thiolated HGCs to enhance the mucoadhesive property of HGC. The ^1^H NMR and ATR-FTIR results confirmed that the chemical modifications, hexanoylation and thiolation of GC were successfully accomplished. The degree of thiolation (DT) of the SH-HGCs could be easily controlled by adjusting the feed molar ratio of 3-mercaptopropionic acid.

The obtained SH-HGC polymers were stored in a refrigerator (below 4 °С) until use. In case that the samples were stored at room temperature for a long time, gel particles were sometimes observed after dispersion in water, presumably due to disulfide bond crosslinking formation. Bernkop-Schnürch et al. synthesized two kinds of thiolated polymers based on polycarbophil and chitosan and studied their stability at several different storage conditions for 6 months [34]. They reported that thiol groups of the thiolated polymers which were stored at room temperature (20 °С) only decreased. Therefore, the thiolated polymers should be stored under adequate conditions (below 4 °С and low humidity) to preserve the thiol groups intact.

Sol-gel transition behaviors of HGC and SH-HGCs were studied by the tube inverting method and rheological measurements. The obtained SH-HGCs had lower T_gel_ values compared to HGC since the residual free amine groups of HGC were substituted with more hydrophobic thiol-propyl amide groups, which may enhance the hydrophobic interaction for thermogelation. In the same way, the SH-HGC having a higher DT (SH10-HGC) showed a lower T_gel_ value relative to one having a lower DT (SH5-HGC). Accordingly, the thermosensitive properties of SH-HGCs could be tuned by controlling the DT of SH-HGC. Rheological study as a function of temperature showed similar results as the above one. GC did not show thermosensitivity while HGC and SH-HGCs showed thermogelling behaviors in the experimental temperature range from 10 to 50 °С. Considering the change of G’ values as a function of temperature, SH10-HGC represented the strongest thermosensitivity among the GC derivatives.

Hassan and Gallo first reported a simple rheological method to assess in vitro mucin-polymer bioadhesive bond strength. They suggested that the interaction between polymer and mucin causes a rheological change [30]. The SH-HGCs showed higher △G` values relative to that of the HGC since SH-HGCs demonstrated a stronger interaction between gel and mucin compared with the HGC. SH-HGCs have both free amine groups and thiol groups on their polymer backbone; thus, these functional groups of SH-HGC might interact with those of mucins by ionic interaction and disulfide covalent bonding [27]. However, the HGC only exhibits ionic interactions, so this noncovalent bond provides only weak interactions with mucin. SH10-HGC had the highest △G`, indicating that more thiol groups in the polymer might cause more efficacious polymer-mucin interactions. Gelation of a polymeric solution might affect the interaction between polymer and mucin. When the aqueous polymeric solutions were gelated, higher △G’s were observed relative to their aqueous solutions (Fig. 5). This supports the idea that the thermosensitive property seems to strengthen the interactions between mucin and the thermogelling polymer by hydrogel formation. Therefore, the obtained SH-HGCs which contain thiol groups and exhibit a thermogelation property have great potential for mucoadhesive applications [35, 36]. Additionally, optimization of the synthesis of SH-HGCs would be required for the applications.

To investigate the potential of such a thermogelling polymer for biomaterial applications, three kinds of in vitro biocompatibility test were accomplished: an MTT assay using HeLa cells and human fibroblasts, the direct contact method using epithelial cells, and live and dead assays using epithelial cell aggregates. In this live and dead assay, we used epithelial cell aggregates to provide an environment similar to the mucosa, which consists of one or more layers of epithelial cells. Generally, a greater number of dead cells are shown in the central region of aggregates because nutrient and oxygen uptake by cells in the inner core of aggregates may be reduced because of the limitation of diffusion [37, 38]. In the case of the SH-HGC, it was observed that reasonable numbers of cells in aggregate were viable. This indicates that the SH-HGCs have little cytotoxicity. The biocompatibility study show that the resultant thiolated polymers are not cytotoxic to several cell lines, including HeLa cells, human fibroblasts, and epithelial cells. Accordingly, the polymers have the potential for mucoadhesive applications.

## Conclusion

In this study, new polysaccharide-based mucoadhesive thermogelling polymers were successfully synthesized by the sequential reactions of *N*-hexanoylation and *N*-thiolation of glycol chitosan. An aqueous solution of HGC and SH-HGCs demonstrated thermosensitive sol-gel transition properties at 4 wt%. SH-HGCs showed a lower transition temperature range compared to HGC due to the hydrophobic thiol group. The rheological mucoadhesion method proved that the covalent attachment of a thiol group to HGC endowed polymers with improved mucoadhesive properties. Cell viability tests showed good biocompatibility of SH-HGCs. Due to their thermogelling property, mucoadhesive property, and low cytotoxicity, SH-HGCs have great potential for biomedical applications.

## Abbreviations

DMSO: Dimethyl sulfoxide
DT: Degree of thiolation
GC: glycol chitosan
HGC: hexanoyl glycol chitosan
MTT: 3-(4,5-dimethylthiazol-2-yl)-2,5-diphenyltetrazolium bromide
SH-HGC: thiolated hexanoyl glycol chitosan
